# Regulation of ryanodine receptor RyR2 by protein-protein interactions: prediction of a PKA binding site on the N-terminal domain of RyR2 and its relation to disease causing mutations

**DOI:** 10.12688/f1000research.5858.1

**Published:** 2015-01-28

**Authors:** Belinda Nazan Walpoth, Burak Erman

**Affiliations:** 1 Swiss Cardiovascular Center, University of Bern, Inselspital, Cardiology, Bern, CH-3012, Switzerland; 2 Department of Chemical and Biological Engineering, Koc University, Instanbul, 34450 S, Turkey

**Keywords:** protein-protein interactions; protein signaling; protein function; ryanodine receptor RyR2; Protein Kinase A; ligand docking; elastic net model; evolutionarily conserved residues; disease causing mutations

## Abstract

Protein-protein interactions are the key processes responsible for signaling and function in complex networks. Determining the correct binding partners and predicting the ligand binding sites in the absence of experimental data require predictive models. Hybrid models that combine quantitative atomistic calculations with statistical thermodynamics formulations are valuable tools for bioinformatics predictions. We present a hybrid prediction and analysis model for determining putative binding partners and interpreting the resulting correlations in the yet functionally uncharacterized interactions of the ryanodine RyR2 N-terminal domain. Using extensive docking calculations and libraries of hexameric peptides generated from regulator proteins of the RyR2 channel, we show that the residues 318-323 of protein kinase A, PKA, have a very high affinity for the N-terminal of RyR2. Using a coarse grained Elastic Net Model, we show that the binding site lies at the end of a pathway of evolutionarily conserved residues in RyR2. The two disease causing mutations are also on this path. The program for the prediction of the energetically responsive residues by the Elastic Net Model is freely available on request from the corresponding author.

## Introduction

**Figure 1.**
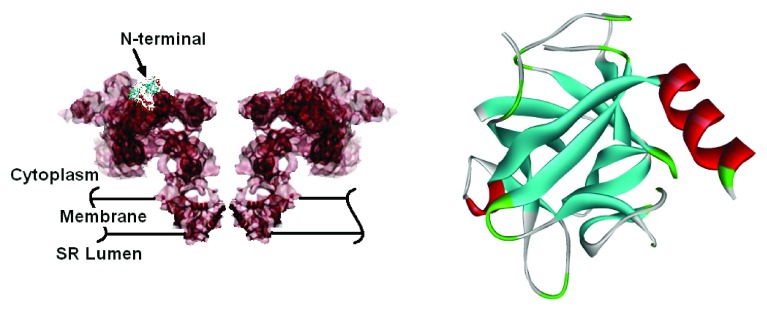
The full structure of RyR2 (5000 residues) is shown in the left panel. The N-terminal region is indicated. The ribbon diagram of the first 217 amino acids of the N-terminal domain is given in the right panel.

Ryanodine receptors are large protein complexes consisting of approximately 5000 residues that form calcium channels that mediate the release of calcium from the sarcoplasmic reticulum, SR, to the cytosol, which is essential for muscle and cardiac rhythm and contractility. There are three forms of ryanodine receptors, RyR1, RyR2 and RyR3. RyR1 is the channel in the skeletal muscle, RyR2 is the type expressed in the heart muscle, and RyR3 is found predominantly in the brain ^1^. The present paper focuses on RyR2. Ca ^++^ release from the SR mediated by RyR2 is a fundamental event in cardiac muscle contraction. These receptors form a group of four homotetramers, with a large cytoplasmic assembly and a transmembrane domain called the pore region. The tridimensional structure of the full assembly is known from cryo-electron microscope studies ^2^ with limited precision. However, the crystal structures of the first 520 amino acids of the N-terminal domain of RyR1 and the first 217 amino acids of the N-terminal domain of the wild type RyR2 and its mutated form are determined with high precision by van Petegem and collaborators ^3^. The main mass of the receptor with dimensions of ca. 280 × 280 × 120 Å is located in the cytoplasmic region, with a stalklike transmembrane region ^2^. The full shape of the channel and the N-terminal are shown in Figure 1.

The cytoplasmic region consists of more than 10 sub-domains that are responsible for the functioning of the receptor through binding to several modulator proteins and ligands ^4^. The modulators include cyclic AMP and protein kinase A (PKA) ^4^, calmodulin ^5^, FKBP12.6 (Calstabin2) ^6^, phosphatases 1 and 2A (PP1 and PP2A) ^7^, sorcin ^8^, and triadin, junctin and calsequestrin ^9^, and several others. Among these, cyclic AMP activates PKA, which in turn phosphorylates RyR2 at SER2809 and SER2815. Despite the important role of the channel, the binding sites of the modulators on the channel are known only approximately. Calmodulin binds to residues located between the positions 3611 and 3642, FKBP12.6 binds to residues around the positions 2361–2496, PP1 around 513 and 808, PP2A around 1451 and 1768, sorcin, triadin, junctin and calsequestrin bind to the vicinity of the transmembrane domain ^7^.

FKBP12.6 binds to RyR2 with a stoichiometry of four FKBP12.6 molecules per single RyR2 channel complex. Binding of FKBP12.6 to RyR2 is required to keep the receptor closed during diastole. In addition to stabilizing individual RyR channels, FKBP12.6 is also required for coupled opening and closing between RyRs. Dissociation of FKBP12.6 from coupled RyR2 channels results in functional uncoupling of the channels leading to heart failure ^4^. Overphosphorylation of RyR2 leads to dissociation of the regulatory protein FKBP12.6 from the channel, resulting in disease ^7^ exhibited as arrhythmias with abnormal diastolic SR Ca++ release. Uncontrolled Ca++ release during the diastole when cytosolic Ca++ concentrations are low can cause delayed after-depolarizations (DADs) which can then lead to fatal arrhythmias. These abnormalities are linked to mutations in the RyR2, located on chromosome 1q42.1–q43 ^10^, which lead to familial polymorphic ventricular tachycardia, CPVT, and arrhythmogenic right ventricular dysplasia type 2, ARVD/C. More than 300 point mutations have been identified in RyR2, some of which are associated with the disorders observed clinically ^11^. In this respect, the N-terminal domain of RyR2, which is known to form an allosteric structure, contains several disease-causing mutations. However, there is yet no information on the mechanisms of the mutations that lead to disease and on the role of these mutations on modulator binding.

None of the modulators discussed above, except PKA, bind to the N-terminal domain. PKA phosphorylates Ser2809 and Ser2815, and it has to anchor to nearby regions of the two serines. PKAs are known to anchor to their hosts at points other than the catalytic domains ^12^. In this study, we generated a hexameric peptide library from the PKA and docked these on several points on the surface of the RyR2 N-terminal. Calculations showed that the hexapeptide PHE LYS GLY PRO GLY ASP from the unstructured C-terminal region of PKA binds to RyR2 with very high affinity, with a dissociation constant of 5.5 nM. For brevity, we will refer to this hexapeptide as the ‘ligand’ and represent it in single letter convention as FKGPGD.

In the last part of the paper, using a coarse grained Elastic Network Model ^13^, we show that the binding site of the ligand lies on a path of energy responsive residues. Energy responsiveness of a residue is defined in terms of correlated fluctuations of that residue with others in the protein. In allosteric proteins, a path of highly correlated residues exists and plays crucial role in energy and signal transfer ^13a, 14^. In RyR2 we identify such a path of highly correlated residues which contains most of the evolutionarily conserved residues. The path also contains the known two disease causing mutations, A77V and R176Q.

## Materials and methods

### Docking predictions

We used the commercial software Gold ^15^ for docking the peptides to the surface of RyR2. The PKA chain (PDB code 2JDV) of 336 amino acids is partitioned into a library of 331 overlapping hexapeptides, such that the first peptide consists of the first six residues 1–6, the second of 2–7, and so on. Several binding sites are chosen on the surface of RyR2 as discussed below. A radius of 20 Å is used for docking. The GoldScore force field is used with rescoring on ChemScore. Flexible docking is used in the first round of calculations. Peptides with reasonable docking energies are chosen after the first run, and a more thorough and extensive docking is performed over this smaller subset. Additional calculations are made with hexapeptide libraries obtained from modulators of RyR2 that are known not to bind at the N-terminal domain as a partial check of the reliability of the method. Optimum binding is obtained for the hexapeptide FKGPGD from the residues 318–323 of 2JDV. The binding energy is obtained as -49 kJ/mol, which is significantly stronger than those of all other investigated hexapeptides. This binding energy corresponds to a dissociation constant *k _D_ = exp(ΔA/kT)* of 5.5 nM.

Our algorithm for the Elastic Net Model uses C ^α^ based coarse graining which evaluates correlations between thermal fluctuations *Δ* $\overset{̭}{R}$ *_i_* and *Δ $\overset{̭}{R}$_j_* in the position of residues i and j. On average, a residue has about eight to 12 neighboring residues to directly interact with. These fluctuation-based interactions are assumed harmonic as if the residues are connected by linear springs. Fluctuations in the distance between two neighboring residues induce changes in their interaction energy. Two residues are assumed neighbors in space if they are closer to each other than a given cutoff distance. This distance corresponds to the radius of the first coordination shell around a given residue, and is usually thought to be between 6.5–7.0 Å. Every pair of residues closer to each other than the cutoff distance is assumed to be connected by a linear spring. The knowledge of the tridimensional structure of the protein that has n residues allows us to write a connectivity matrix, C, where the rows and the columns identify the residue indices, from 1 to n, where the amino-end is the starting and the carboxyl-end is the terminating-end of the protein. If two residues i and j are within the cutoff distance, then *C _ij_* = 1, otherwise it is zero. Another matrix, *Γ _ij_*, is obtained from the connectivity matrix as

$$\Gamma_{\text{ij}}=\left\{ \begin{array}{ccc} –\gamma C_{\text{ij}} & & \text{if i}\neq j\\ –\sum_{k}C_{\text{ik}} & & \text{if i}=j \end{array}\right.\;\;\;\;\;(1)$$

Where *γ* is the spring constant of the harmonic interactions. The relationship of the forces to the displacements is given by the equation *ΔF _i_ =* $\underset{j}{\Sigma }$ *Γ _ij_ΔR _j_*. Techniques of statistical mechanics allow us to derive several relationships between the fluctuations of residues ^16^. The correlation between the fluctuations of residues i and j is related, for example, to the inverse of the matrix *Γ _ij_* as

$$\langle \Delta R_{i}\Delta R_{j}\rangle =k_{B}{T(\Gamma^{\text{-1}})}_{\text{ij}}\;\;\;\;\;(2)$$

Here, the angular brackets denote the time average of the product of fluctuations of residues i and j, *k _B_* is the Boltzmann constant, *T* is the physiological temperature expressed in Kelvin scale, *Γ* ^-1^ is the inverse matrix *Γ*, and its subscripts i and j acknowledge the residue indices of interest. If i = j, then Equation 2 becomes

$$\langle {\left(\Delta R_{i}\right)}^{2}\rangle =k_{B}{T(\Gamma^{\text{-1}})}_{\text{ii}}\;\;\;\;\;(3)$$

The left hand side of Equation 3 is the mean-square fluctuations of the i’th residue which is related to experimentally available B-factors, *B _i_*, through the equation

$$\langle {\left(\Delta R_{i}\right)}^{2}\rangle =\frac{3}{8\pi^{2}}B_{i}\;\;\;\;\;(4)$$

The mean-square fluctuations 〈( *ΔR _ij_*) ^2^〉 of the distance *ΔR _ij_* between residues i and j are obtained from Equation 3 as

$$\langle {\left(\Delta R_{i}\right)}^{2}\rangle ={(\Gamma^{\text{–1}})}_{\text{ii}}–2{(\Gamma^{\text{–1}})}_{\text{ij}}+{(\Gamma^{\text{–1}})}_{\text{jj}}\;\;\;\;\;(5)$$

The derivation of Equation 5 is given in the reference ^17^. Calculations presented therein showed that the largest few eigenvalues of *Γ* give information at the residue level. Smaller eigenvalues are associated with global motions. Our calculations showed that the largest five eigenvalues and the corresponding eigenvectors are satisfactory for representing fluctuations at the residue level.

Fluctuations of the harmonic energy between two residues are proportional to the mean square fluctuations of the distance between the two. Thus, Equation 5 is representative of energy fluctuations, and summing over all the neighbors of the residue i shows the energy response *ΔU _i_* of residue i with its surroundings:

$$\Delta U_{i}\sim \sum_{j}\langle {\left(\Delta R_{\text{ij}}\right)}^{2}\rangle \;\;\;\;\;(6)$$

This is a thermodynamically meaningful quantity showing the mean energy response of residue i to all fluctuations of its surroundings. These correlations extend throughout the protein, leading to specific paths along which the fluctuations propagate. Recent work shows that these paths are evolutionarily conserved ^14a^.

The N-terminal domain of RyR2 is a signal protein of 217 amino acids. The crystal structure of the N-terminal domain of physiological RyR2 (PDB code 3IM5) and the A77V mutated crystal structure (PDB code 3IM7) have been determined by x-ray with resolutions of 2.5 and 2.2 Å, respectively, by Van Petegem and Lobo ^3a^. The protein consists of a β-trefoil of 12 β strands held together by hydrophobic forces. A 10-residue α helix is packed against strands β4 and β5. A 3 residue 3–10 helix is present in the loop containing β3 and β4. The N-terminal contains two MIR domains, similar to the inositol 1,4,5-triphosphate receptor (IP3R), for which ligand-induced conformational changes have been studied more extensively ^18^.

## Results and discussion

### Docking results

**Figure 2.**
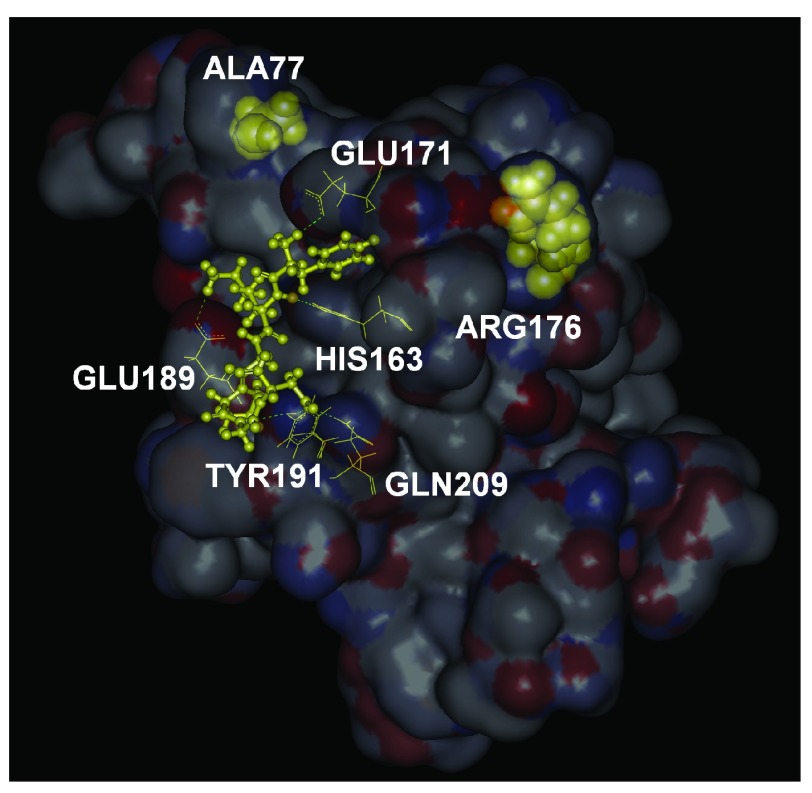
The bound conformation of FKGPGD, shown in yellow ball and stick. Residues with which it forms hydrogen bonds are shown in yellow wire, and labeled. The two disease causing mutation residues, ALA77 and ARG176 are shown in yellow CPK.

The binding free energy of FKGPGD to the surface shown in Figure 2 is obtained as -49 kJ/mol by the ChemScore potential, which corresponds to a dissociation constant of 5.5 nM. The 42% of the binding energy comes from hydrogen bonds and 39% from lipophilic interactions. The dissociation constant of 5.5 nM is at least two orders of magnitude better than the values obtained for the other hexapeptides of the library. It is therefore highly likely that PKA anchors itself on RyR2 at the position shown.

### The energy conduction path of RyR2

**Figure 3.**
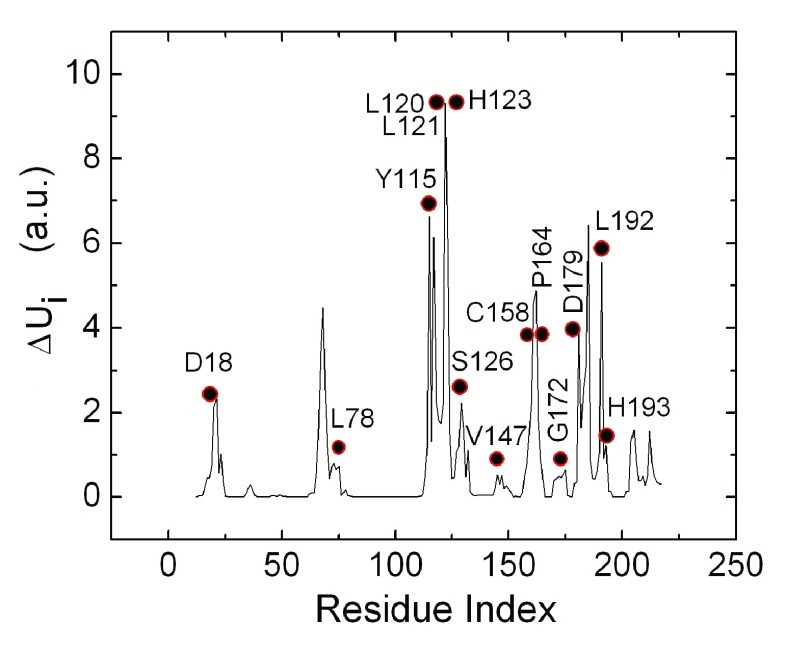
Energetically responsive residues (solid line) obtained with the Elastic Net Model, and the conserved residues (circles) obtained from Reference 22. In Reference 20, conservation levels are ordered from 1 to 8, the latter being the highest degree of conservation. The filled circles correspond to residues with level 8. The ordinate values are in arbitrary un-normalized units.

In order to interpret the binding of the PKA on RyR2, we performed elastic net analysis of energetically responsive residues of RyR2. The residues that yield high values of the energy response defined by Equation 6 are calculated according to the scheme outlined in the Methods section. In Figure 3, the mean energy response *ΔU _i_* of residue i is presented along the ordinate as a function of residue index. The circles indicate the highest conserved residues of 3IM5, obtained from the work of Goldenberg *et al.* (See also the PDBSum web site ^22^).

Comparison of the solid curve peaks and the circles shows that there is a strong correlation between the energy responsive and conserved residues, in agreement with the recent suggestion of Lockless and Ranganathan ^14a^. The set of conserved residues, with the highest level of conservation according to Reference 20 of the protein, all lie within the set of energetically responsive residues and are located along or in the neighborhood of the path obtained from the energetically responsive residues. On the three-dimensional structure of the protein, the peaks shown in Figure 3 constitute a path of residues that are spatial neighbors.

A residue or set of residues at the surface of the protein which are energy responsive are expected to be the hotspots for binding, because these residues can exchange energy with the surroundings, and distribute the energy taken from the surroundings to the other residues of the protein. According to this conjecture, one needs to dock ligands only to the hotspots identified with the peaks in Figure 3. In our calculations, we adopted five such hotspot regions for docking. These hotspot regions are centered at: (1) VAL21, (2) VAL68, (3) ARG122, (4) SER185, and (5) ALA205. In the complex structure of the channel, some of these five surface regions may not be exposed to ligands but may be facing the other domains of the channel. However, a residue that neighbors another domain may become exposed to a ligand upon opening of the channel. We carried out the calculations for the five regions stated above, irrespective of their neighborhood.

**Figure 4.**
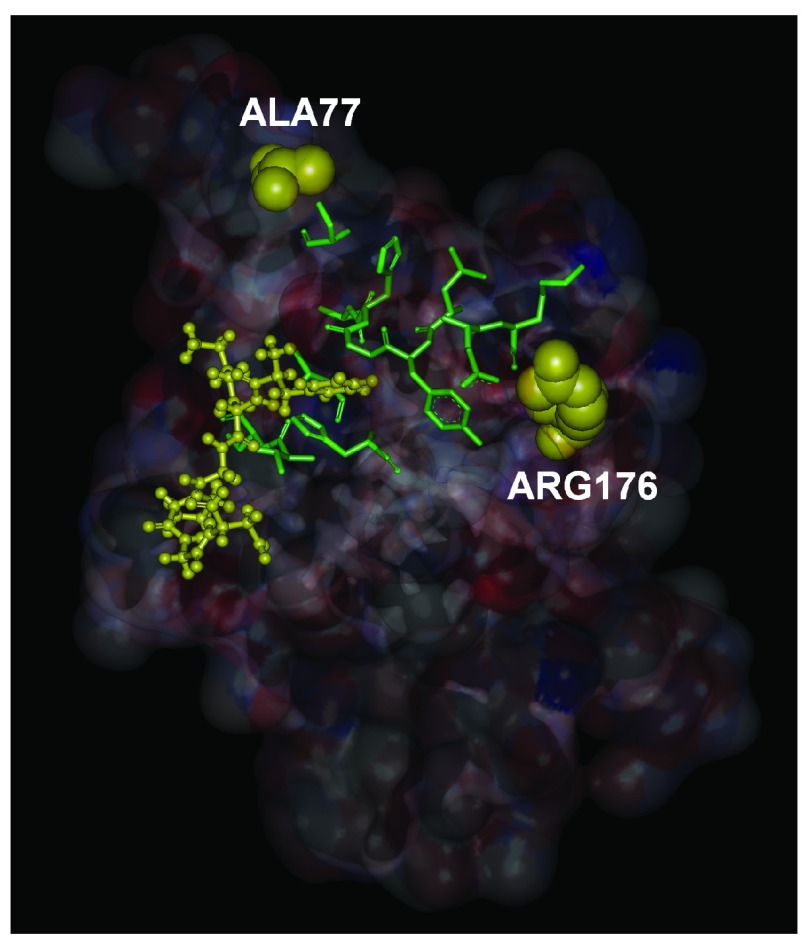
Energy interaction paths from ALA77 and ARG176 to the ligand. The residues shown in stick form are conserved residues which are also identified by the peaks in Figure 3. The hexamer ligand is shown in ball and stick form.

In Figure 4, we show, in stick form, the evolutionarily highly conserved residues that lie along a path between ALA77, ARG176 and the ligand FKGPGD of PKA. The peak residues clearly form a path between the ligand and the mutation residues. The path shown in the figure contains the energetically responsive residues predicted by the GNM as may be seen from Figure 3. Using extensive docking calculations and libraries of residues obtained from regulator proteins of the RyR2 channel, we showed that residues 318–323 of PKA have a very high affinity for the N-terminal of RyR2. The location of binding is a pocket bordered by GLU171 and GLU189. GLU171 is a conserved residue and participates in calcium binding in inositol 3 receptors, IP3R. However, a ligand for RyR2 at GLU171 is not yet known. We also showed that the disease causing mutations ALA77VAL and ARG176GLN are joined by an energy interaction pathway to the ligand binding surface. Although these two mutations are responsible for arrhythmias, their exact mechanism is not known. The present model directs attention to the relationship between the residues at the binding site, the predicted path of energy responsive residues and the two disease causing mutation sites. Since binding of PKA to RyR2 results in phosphorylation of the latter, and since hyperphosphorylation leads to disease, one may indirectly conjecture that mutations in the two residues modify the binding characteristics of PKA.

### Relative orientations of RyR2 and PKA in bound form

**Figure 5.**
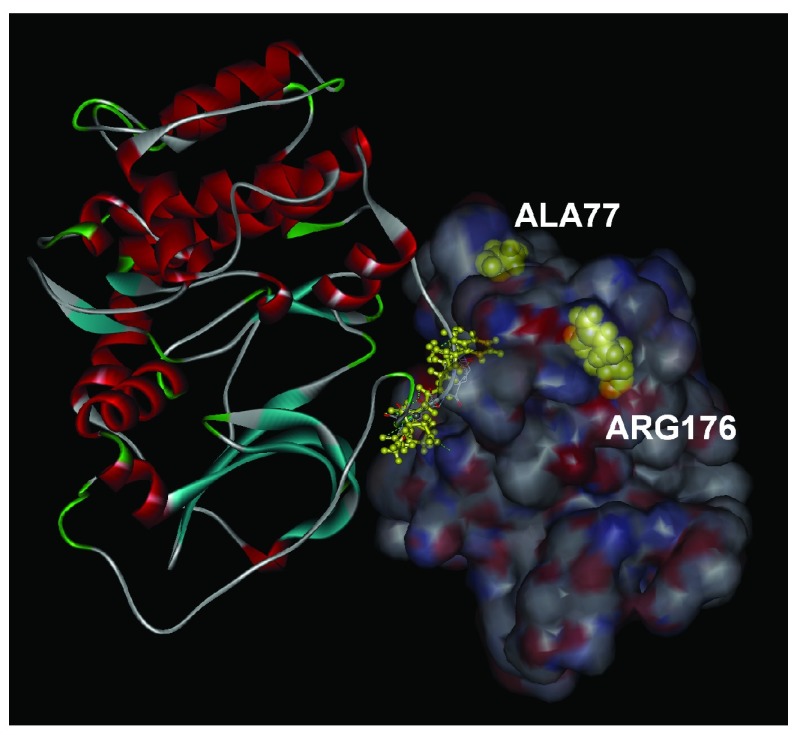
Relative orientations of RyR2, shown in surface, and PKA, shown in solid ribbon. The sequence FKGPGD of PKA is shown in ball and stick form.

Superposition of the three dimensional PDB structures of PKA and RyR2 in such a way that the residues FKGPGD of PKA are kept in the bound state gives the relative orientations of the two proteins. This is shown in Figure 5.

Using the Elastic Net Model, we identified the energy conduction pathway for the wild type RyR2. This path whose residues are shown in Figure 3 has several features of interest. Firstly, it contains most of the evolutionarily conserved residues. The remaining conserved residues are in the close neighborhood of the path, all making hydrogen bonds with the residues of the path. This important feature has recently been shown by Lockless and Ranganathan ^14a^ implying that evolutionary conservation is driven by energy conduction in proteins. Although no ligands for the RyR2 N-terminal have been observed until now ^19^, the three glutamic acids, GLU171, GLU173 and GLU189 at the pocket may potentially form a binding site. This suggestion is also based on the observation that in IP3R a potential calcium binding site is formed by GLU283 and GLU285 whose location on the path coincides exactly with that of RyR2. The residue GLU173 is exposed to water and is a candidate for possible binding.

The underlying determinant of the allosteric pathway, defined as the path of energy responsive residues in the present paper, is the graph structure of the protein ^20^. The view that proteins relay signals by energy fluctuations in response to inputs, have been recently discussed in an elegant paper by Smock and Gierasch ^14b^. In the present study, we showed that evolutionarily conserved residues lie on the pathway of energy responsive residues. RyR2 contains two interspersed MIR domains, residues 124–178 and 164–217 ^21^. Elastic net calculations show that the residues that lie on the energy conduction pathway are closely associated with these MIR domains: the energy responsive residues either lie on the MIR domains, or they are hydrogen bonded to the residues of these domains. There is no energetically responsive residue that is not closely associated with the MIR domain. We therefore conclude that the MIR domains of RyR2 play an active role in energy transport through the protein.

Data of predicted PKA binding sites on RyR2 Highly conserved residues that lie along a path between ALA77, ARG176 and the ligand FKGPGD of PKA are shown. Amplitude for each residue is indicated.

## Data availability

Data of predicted PKA binding sites on RyR2 ^23^.

## References

[1] Kimlicka L Van Petegem F: The structural biology of ryanodine receptors. *Sci China Life Sci.* 2011;54(8):712–724. 10.1007/s11427-011-4198-2 21786194

[2] Hamilton SL Serysheva II: Ryanodine receptor structure: progress and challenges. *J Biol Chem.* 2009;284(7):4047–4051. 10.1074/jbc.R800054200 18927076 PMC3837402

[3a] Lobo PA Van Petegem F: Crystal structures of the N-terminal domains of cardiac and skeletal muscle ryanodine receptors: insights into disease mutations. *Structure.* 2009;17(11):1505–1514. 10.1016/j.str.2009.08.016 19913485

[3b] Lobo PA Kimlicka L Tung CC: The deletion of exon 3 in the cardiac ryanodine receptor is rescued by β strand switching. *Structure.* 2011;19(6):790–798. 10.1016/j.str.2011.03.016 21645850

[3c] Tung CC Lobo PA Kimlicka L: The amino-terminal disease hotspot of ryanodine receptors forms a cytoplasmic vestibule. *Nature.* 2010;468(7323):585–588. 10.1038/nature09471 21048710

[4] Wehrens XH Marks AR: Altered function and regulation of cardiac ryanodine receptors in cardiac disease. *Trends Biochem Sci.* 2003;28(12):671–678. 10.1016/j.tibs.2003.10.003 14659699

[5] Fruen BR Bardy JM Byrem TM: Differential Ca(2+) sensitivity of skeletal and cardiac muscle ryanodine receptors in the presence of calmodulin. *Am J Physiol Cell Physiol.* 2000;279(3):C724–C733. 10942723 10.1152/ajpcell.2000.279.3.C724

[6a] Marx SO Gaburjakova J Gaburjakova M: Coupled gating between cardiac calcium release channels (ryanodine receptors). *Circ Res.* 2001;88(11):1151–1158. 10.1161/hh1101.091268 11397781

[6b] Brillantes AB Ondrias K Scott A: Stabilization of calcium-release channel (ryanodine receptor) function by Fk506-binding protein. *Cell.* 1994;77(4):513–523. 10.1016/0092-8674(94)90214-3 7514503

[7] Marx SO Reiken S Hisamatsu Y: PKA phosphorylation dissociates FKBP12.6 from the calcium release channel (ryanodine receptor): defective regulation in failing hearts. *Cell.* 2000;101(4):365–376. 10.1016/S0092-8674(00)80847-8 10830164

[8] Meyers MB Pickel VM Sheu SS: Association of sorcin with the cardiac ryanodine receptor. *J Biol Chem.* 1995;270(44):26411–26418. 10.1074/jbc.270.44.26411 7592856

[9] Zhang L Kelley J Schmeisser G: Complex formation between junction, triadin, calsequestrin, and the ryanodine receptor. Proteins of the cardiac junctional sarcoplasmic reticulum membrane. *J Biol Chem.* 1997;272(37):23389–23397. 10.1074/jbc.272.37.23389 9287354

[10] Wang J Prakasa K Bomma C: Comparison of novel echocardiographic parameters of right ventricular function with ejection fraction by cardiac magnetic resonance. *J Am Soc Echocardiogr.* 2007;20(9):1058–1064. 10.1016/j.echo.2007.01.038 17555927

[11a] Kimlicka L Van Petegem F: The structural biology of ryanodine receptors. *Sci China Life Sci.* 2011;54(8):712–724. 10.1007/s11427-011-4198-2 21786194

[11b] Betzenhauser MJ Marks AR: Ryanodine receptor channelopathies. *Pflugers Arch.* 2010;460(2):467–480. 10.1007/s00424-010-0794-4 20179962 PMC2885589

[11c] Priori SG Chen SR: Inherited dysfunction of sarcoplasmic reticulum Ca2+ handling and arrhythmogenesis. *Circ Res.* 2011;108(7):871–883. 10.1161/CIRCRESAHA.110.226845 21454795 PMC3085083

[11d] Thomas NL Maxwell C Mukherjee S: Ryanodine receptor mutations in arrhythmia: The continuing mystery of channel dysfunction. *FEBS Lett.* 2010;584(10):2153–2160. 10.1016/j.febslet.2010.01.057 20132818

[12] Huang LJ Durick K Weiner JA: Identification of a novel protein kinase A anchoring protein that binds both type I and type II regulatory subunits. *J Biol Chem.* 1997;272(12):8057–8064. 10.1074/jbc.272.12.8057 9065479

[13a] Erman B: Relationships between ligand binding sites, protein architecture and correlated paths of energy and conformational ﬂuctuations. *Phys Biol.* 2011;8(5):056003. 10.1088/1478-3975/8/5/056003 21832802

[13b] Haliloglu T Erman B: Analysis of correlations between energy and residue fluctuations in native proteins and determination of specific sites for binding. *Phys Rev Lett.* 2009;102(8):088103. 10.1103/PhysRevLett.102.088103 19257794

[13c] Haliloglu T Seyrek E Erman B: Prediction of binding sites in receptor-ligand complexes with the Gaussian Network Model. *Phys Rev Lett.* 2008;100(22):228102. 10.1103/PhysRevLett.100.228102 18643462

[14a] Lockless SW Ranganathan R: Evolutionarily conserved pathways of energetic connectivity in protein families. *Science.* 1999;286(5438):295–299. 10.1126/science.286.5438.295 10514373

[14b] Smock RG Gierasch LM: Sending signals dynamically. *Science.* 2009;324(5924):198–203. 10.1126/science.1169377 19359576 PMC2921701

[15] http://www.ccdc.cam.ac.uk/products/life_sciences/gold/.

[16a] Haliloglu T Erman B: Analysis of correlations between energy and residue fluctuations in native proteins and determination of specific sites for binding. *Phys Rev Lett.* 2009;102(8):088103–088106. 10.1103/PhysRevLett.102.088103 19257794

[16b] Yogurtcu ON Gur M Erman B: Statistical thermodynamics of residue fluctuations in native proteins. *J Chem Phys.* 2009;130(9):095103–13. 10.1063/1.3078517 19275429

[16c] Haliloglu T Gul A Erman B: Predicting important residues and interaction pathways in proteins using Gaussian Network Model: binding and stability of HLA proteins. *PLoS Comput Biol.* 2010;6(7):e1000845. 10.1371/journal.pcbi.1000845 20628622 PMC2900293

[17] Tuzmen C Erman B: Identification of ligand binding sites of proteins using the Gaussian Network Model. *PLoS One.* 2011;6(1):e16474. 10.1371/journal.pone.0016474 21283550 PMC3026835

[18a] Lin CC Baek K Lu Z: Apo and InsP _3_-bound crystal structures of the ligand-binding domain of an InsP _3_ receptor. *Nat Struct Mol Biol.* 2011;18(10):1172–1174. 10.1038/nsmb.2112 21892169 PMC3242432

[18b] Yuchi Z Van Petegem F: Common allosteric mechanisms between ryanodine and inositol-1,4,5-trisphosphate receptors. *Channels (Austin).* 2011;5(2):120–123. 10.4161/chan.5.2.14313 21150295

[19] van Petegem F: Private correspondence.

[20] Rader AJ Brown SM: Correlating allostery with rigidity. *Mol Biosyst.* 2011;7(2):464–471. 10.1039/c0mb00054j 21060909

[21] Amador FJ Liu S Ishiyama N: Crystal structure of type 1 ryanodine receptor amino-terminal beta-trefoil domain reveals a disease-associated mutation “hot spot” loop. *Proc Natl Acad Sci U S A.* 2009;106(27):11040–11044. 10.1073/pnas.0905186106 19541610 PMC2708722

[22] PDBSUM. Reference Source

[23] Erman B Walpoth BN Erman B: Data of predicted PKA binding sites on RyR2. *figshare.* 2015 Data Source

