# Supplementary material for: Regulation of ryanodine receptor RyR2 by protein-protein interactions: prediction of a PKA binding site on the N-terminal domain of RyR2 and its relation to disease causing mutations
Source: F1000Res. 2015 Jan 28;4:29. [Version 1] doi: 10.12688/f1000research.5858.1 (PMC4392826; doi:10.12688/f1000research.5858.1)
Supplement: Peer review history

# Peer review: Referee response for version 1

**DOI:** 10.5256/f1000research.6260.r8179

**Published:** 2015-04-01

**Authors:** Pemra Doruker

**Affiliations:** 1 Department of Chemical Engineering and Polymer Research Center, Bogazici University, İstanbul, Turkey

This is an interesting paper that utilizes alternative methodologies for protein-protein docking. Especially determination of energy responsive residues- hotspots for binding- based on elastic network model deserves attention as it has been previously assessed in terms of ligand binding by Tuzmen and Erman (2011). However, the peptide docking procedure, which has detected a six-residue long peptide ‘FKGPGD’ located on an unstructured loop, is a non-standard approach as far as to my knowledge. The authors need to discuss why they have preferred such a technique for their problem and specifically used six-residue peptides. More details on the docking procedure/steps and efficiency would also be helpful.  Further validation of the resulting complex by a standard protein-protein docking algorithm would also be welcome.

I have read this submission. I believe that I have an appropriate level of expertise to confirm that it is of an acceptable scientific standard.

# Peer review: Referee response for version 1

**DOI:** 10.5256/f1000research.6260.r8042

**Published:** 2015-03-19

**Authors:** Ugo Bastolla

**Affiliations:** 1 Centro de Biologia Molecular Severo Ochoa, CSIC-UAM, Madrid, Spain

The Ryanodine Receptor RyR2 is a key transporter of calcium in the heart, and some of its mutations are associated with fatal arrhythmias. In this paper, RyR2 is studied through docking and elastic network model (ENM) computations. The authors find that a hexa-peptide of the PKA kinase, a known regulator of RyR2, has a very high affinity for RyR2, in the nano-molecular range, and that its binding site is connected to two known disease-associated residues (A77 and R176) through a “path” of residues predicted to be “energy responsive” through ENM calculations. Furthermore, energy responsive residues are strongly conserved.

I think that the paper is very interesting, although some aspects of its presentation have to be improved, in particular concerning the interesting theoretical part on energy responsiveness.

I tend to agree with the other reviewer that such a large affinity between the PKA kinase and RyR2 is strange, since kinase interactions are expected to be transient. However, I think that it is unlikely that such a high affinity arises by chance. If we assume that it is correct, it may suggest a molecular mechanism for the disease, as follows. After phosphorylation, the kinase has to dissociate, which would be very difficult given the high affinity, unless a conformation change takes place upon phosphorylation and triggers the release of PKA. This mechanism may be consistent with the network of highly dynamic residues predicted by the ENM, which connects the binding site and the disease-associated site. If the mutations perturb the dynamics of RyR2, making the release of the kinase more difficult, they would result in overphosphorylation, which is the known signature of the disease. It would be interesting to investigate this hypothesis through ENM (and/or MD) computations that try to connect the phosphorylation site and the binding site of the kinase through a signaling pathway and test whether this pathway passes through the disease associated residues, as the computations presented here may suggest.

Concerning the presentation, the result that energy-responsive residues are strongly conserved is very interesting. The authors relate it with the influential proposal by Lockless and Raganathan (LR) that pairs of residues energetically coupled are evolutionarily coupled as well (actually this field of correlated mutations is quite older than the LR article and it recently experienced an explosion of interest), but the LR proposal is not exactly the same as what is found here, since in this paper conservation of individual residues rather than evolutionarily correlations are observed. Thus, it would be interesting to study whether this correlation between responsiveness and conservation is general, or it is a special property of RyR2 and maybe other strongly allosteric proteins. Of course I do not require that the authors do this study in this paper, but it would be useful that they share their thoughts on this point. Since the responsiveness of a residues must be correlated with its intrinsic fluctuations (predicted B factors), it would be interesting to known whether the former or the latter correlates more strongly with conservation.

Furthermore, the authors expect that responsive residues are hotspots of binding. This expectation does not seem so natural to me. Actually, I expected almost the opposite: two residues i and j that bind the same molecule must have small fluctuations of their interatomic distance <(DeltaR_ij)^2>, so that I expected that residues in a binding site must be characterized by <(DeltaR_ij)^2> smaller than generic residues. I tested this expectation some time ago, finding that it is true on the average but much weaker than I expected. Thus, the fact that responsive residues are close to binding sites can be interpreted either as a general property or as a special property of allosteric proteins in which binding and conformation changes are related. It would be interesting to discuss it.

Finally, although the paper is very well written, there are some small typos in the equations: 1) In Eq.(1) the factor gamma has to appear in the second line as well; 2) Temperature has disappeared from Eq.(5) ; 3) between Eq.(5) and (6), it is said that the five largest eigenvalues are enough for representing fluctuations, I think that the authors mean “smallest eigenvalues”, and it would be useful to say that the number five holds for RyR2, other proteins may have a different number of relevant modes

I have read this submission. I believe that I have an appropriate level of expertise to confirm that it is of an acceptable scientific standard.

# Peer review: Referee response for version 1

**DOI:** 10.5256/f1000research.6260.r7520

**Published:** 2015-02-04

**Authors:** Xiayang Qiu

**Affiliations:** 1 Structural Biology and Biophysics, Worldwide Research and Development, Pfizer Inc., Groton, CT, USA

In this manuscript, the authors first performed computational docking studies to suggest that a hexapeptide of PKA has a high-affinity binding to the N-terminal domain of RyR2. Then, based on the elastic network model calculations, the authors argued that the putative PKA binding site on the N-terminal domain of RyR2 lies at the end of an energy conduction pathway of conserved residues.  While such insights would be very important for understanding the biology of RyR2, there are a few serious points needed to be addressed prior to indexing, mostly due to the lack of experimental data to sustain the authors' claims. 

1. The author had made the assumption that the binding epitope of PKA would be a linear peptide. Why wouldn't it be a 3D structural binding epitope with contributions from residues close in space but not close in sequence?
2. The author suggested that the hexapeptide would bind to the N-terminal domain of RyR2 with an affinity of 5.5nM, which is quite high given the relatively small and flat interface. Considering the interaction is more likely to be transient than permanent *in vivo,* the authors need to provide more evidences or references to either support the argument or soften the affinity claim as a theoretical prediction or hypothesis.
3. If the hexapeptide, FKGPGD, is indeed showing such a high affinity to the N-terminal domain of RyR2 how unique is such a hexapeptide among all proteins? Would other RyR2 modular proteins share a similar sequence? Or would other proteins sharing such a hexapeptide modulate the function of RyR2?
4. Regarding the phosphorylation sites by PKA, based on a number of references (For example, UniProt database http://www.uniprot.org/uniprot/Q92736), RyR2 is phosphorylated by PKA at two major sites, Ser2031 (Ser2030 in certain publications) and Ser2808 (Ser2809 in certain publications). On the other hand, Ser2814 (Ser2815 in certain publications) is mainly phosphorylated by CaMK2D. This would be consistent with the fact that PKA phosphorylates proteins that have the motif Arg-Arg-X-Ser exposed. In the manuscript, the authors suggested that PKA phosphorylates RyR2 at Ser2809 and Ser2815; however, Ser2815 does not seem to fit such a recognition pattern of PKA.
5. Since experimentally PKA is known to phosphorylate RyR2 at two different sites, does such a high-affinity anchor model fit in the experimental observations?

Based on this, we suggest the author re-visit the manuscript to either provide some kind of experimental evidence, or tone down some of the biological implications - focusing rather on the computation approaches and the intriguing results thereof that may or may not be true in the end.

I have read this submission. I believe that I have an appropriate level of expertise to confirm that it is of an acceptable scientific standard, however I have significant reservations, as outlined above.
